# Microbial activity in Martian analog soils after ionizing radiation: implications for the preservation of subsurface life on Mars

**DOI:** 10.3934/microbiol.2018.3.541

**Published:** 2018-07-09

**Authors:** Vladimir S. Cheptsov, Elena A. Vorobyova, George A. Osipov, Natalia A. Manucharova, Lubov' M. Polyanskaya, Mikhail V. Gorlenko, Anatoli K. Pavlov, Marina S. Rosanova, Vladimir N. Lomasov

**Affiliations:** 1 Soil Science Faculty, Lomonosov Moscow State University, Moscow, Russia; 2 Space Research Institute, Russian Academy of Sciences, Moscow, Russia; 3 International Analytical Center, Interlab, N.D.Zelinsky Institute of Organic Chemistry, Moscow, Russia; 4 Ioffe Physical-Technical Institute, Russian Academy of Sciences, St. Petersburg, Russia; 5 Peter the Great St. Petersburg State Polytechnic University, St. Petersburg, Russia

**Keywords:** astrobiology, Mars, gamma radiation, microbial communities, radioresistance

## Abstract

At present, the surface of Mars is affected by a set of factors that can prevent the survival of Earth-like life. However, the modern concept of the evolution of the planet assumes the existence more favorable for life climate in the past. If in the past on Mars had formed a biosphere, similar to the one that originated in the early Earth, it is supposed that it is preserved till now in anabiotic state in the bowels of the planet, like microbial communities inhabiting the ancient permafrost of Arctic and Antarctic. In the conditions of modern Martian regolith, this relic life seems to be deprived of the possibility of damage reparation (or these processes occur on a geological time scale), and ionizing radiation should be considered the main factor inhibiting such anabiotic life. In the present study, we studied soil samples, selected in two different extreme habitats of the Earth: ancient permafrost from the Dry Valleys of Antarctica and Xerosol soil from the mountain desert in Morocco, gamma-irradiated with 40 kGy dose at low pressure (1 Torr) and low temperature (−50 °C). Microbial communities inhabiting these samples showed *in situ* high resistance to the applied effects, retained high number of viable cells, metabolic activity, and high biodiversity. Based on the results, it is assumed that the putative biosphere could be preserved in the dormant state for at least 500 thousand years and 8 million years in the surface layer of Mars regolith and at 5 m depth, respectively, at the current level of ionizing radiation intensity.

## 1. Introduction

Mars is one of the most promising objects for intensive astrobiological research in the Solar System. Mars conditions are a complex of physico-chemical factors that are generally considered to be extreme with respect to the only known terrestrial life form and, apparently, are capable of hindering its origin and preservation in a viable state. These extreme factors are low temperature, predominantly carbon dioxide atmosphere with low pressure, the presence of strong oxidants, intense ultraviolet and ionizing radiation, and deficiency of liquid water [1]–[3].

However, the scientific concept of the evolution of the planet neighboring Earth presupposes the existence of more favorable for life climate in the past [4]–[7]. The duration of this period is unknown. Nevertheless, if the biosphere, similar to the one that very quickly has originated on the early Earth [8],[9] has arisen at early stage of Mars evolution, it is supposed that it is still preserved in a cryptic anabiotic state in subsurface of the planet [4],[10]. The basis for this hypothesis is the research on the detection of life in the ancient permafrost of Antarctica [11],[12], evaporites, cryopegs [13] and other extreme habitats. In the conditions of modern Martian regolith, such a relic life seems to be deprived of the possibility of damage reparation or these processes occur on a geological time scale, and ionizing radiation should be considered the main factor inhibiting the dormant biosphere [14]. Thus, if we assume the possibility of the biosphere formation on early Mars, it is necessary to answer the question of the possible duration of cryptic life survival in Martian conditions after losing significant part of atmosphere.

An approximate estimation could be given on the basis of known data on the radioresistance of terrestrial organisms. However, it must be taken into account that physical conditions can significantly change the amount of radiation damages in cells. It is known, that the most of radiation damages in the cell under gamma irradiation at normal conditions are caused by highly reactive oxygen species like free radicals OH, O_2_H, etc. [15]–[17]. Irradiation conditions including temperature, pressure, atmosphere composition, and water content could significantly affect free radicals amount and mobility. Water and oxygen are considered like the most important sources of free radicals at irradiation. Because that water content in the cells and oxygen concentration in the atmosphere could directly influence free radicals amount [15],[16]. It was shown, that desiccated microbial cells survive higher irradiation doses than cells in suspensions [18],[19]. Low atmospheric pressure must lead to the cells desiccation, and subsequently, increase radioresistance. Temperature conditions during irradiation impact free radicals mobility. It was shown that free radicals such as OH and O_2_H, generated at radiolysis of the water are stable at −196 °C and do not diffuse to attack substrate molecules [20]. At temperatures above −153 °C these radicals can diffuse and chemically attack molecules, but their mobility is restricted depending on the temperature [21]. Dependence of bacteria irradiation survival with temperature conditions during irradiation was repeatedly observed [21]–[23]. In addition, there are evidences that composition of organo-mineral matrix composition in which cells are irradiated can influence irradiation effects. Such data are contradictory and the issue is insufficiently studied [24]–[26]. Due to the foregoing facts, for an accurate forecast of the radioresistance of microorganisms in extraterrestrial conditions it is necessary to model the joint impact of the most complete possible set of factors.

To predict the longevity of the putative biosphere existence, it is necessary to study the stability of natural homeostatic microbial systems, rather than individual microbial isolates, whose physiological responses to stress change significantly after extraction from the native habitat into culture. It is important to estimate the ability of natural microbial communities not only to preserve any cells counts, but to conserve biodiversity and implement their biospheric functions (i.e. to conduct biogeochemical processes). Microbial communities of extreme habitats are considered as terrestrial analogs of objects of astrobiological search for extant life. They are adapted to existence under extreme conditions (as, probably, putative organisms on Mars). Such analogues are the ecosystems of the Arctic and Antarctic permafrost and ices, desert and saline soils, and others [27]–[31]. It is important to study the sustainability and metabolism of such communities in their natural habitats, interaction with which is necessary for long-term maintenance of viability. The protective role of the natural organo-mineral environment and intra- and interpopulation interactions should be taken into account, which substantially corrects the stability of native biosystems [26],[32]–[35].

In the present study, we have irradiated soil and permafrost samples from extreme habitats with gamma radiation at 40 kGy dose under low pressure (1 Torr) and low temperature (−50 °C) conditions. Microbiological analysis was carried out to estimate the possible duration of cryoconservation of putative Earth-like microbial communities in Martian regolith in dormant state.

## 2. Materials and methods

### 2.1. Samples description

Antarctic permafrost (sample A-6/99-6) and a sample of Xerosol soil sampled in the mountain desert at the foot of the Atlas, Morocco (sample S1) were used for the study.

Sample A-6/99-6 was taken from 1.3–1.5 m depth in 6/99 well drilled in the flat area of the Beacon Valley (Antarctic, Dry Valleys, 77°50′S, 160°36′E, 1270 m above sea level) [12]. According to various estimates, the age of the permafrost ranges from 50–300 thousand years to 8.1 million years [12],[36]. Sedimentary rock is a coarse-grained sand with inclusions of pebbles cemented by ice into a massive cryogenic structure with a depth of at least 20 m (hereinafter, not drilled). The pH of the precipitation is predominantly alkaline (7.8–9.8). Eh, measured by a combined platinum electrode at 20 °C in thawed suspensions, varied between +260 to +480 mV, indicating aerobic conditions. Ions concentrations were determined using Dionex ICS-1100 Ion Chromatography System (“Dionex Corporation”, USA) according manufacturer recommendations; total organic carbon (TOC) content was determined using method of oxidation in potassium dichromate [37]. The TOC content was very low (no more than 0.01%). The chemical composition of the aqueous extracts showed the dominance of the cations Na^+^ and Mn^2+^ and the anions Cl^−^ and CO_3_^2−^ (Table 1). The concentrations of O_2_ (18.9–20.6%) and N_2_ (75.5–77.2%) in frozen sediments were similar to those in the air. The maximum negative temperature recorded in the permafrost samples studied is −18.5 °C. The method of sampling and transporting it to the laboratory was eliminating melting and was described earlier [12]. After delivery to the laboratory, the sample was stored at −18 °C.

**Table 1.** Chemical characteristic of the samples.

Sample	рН	NO_2_^−^, mg/kg	NO_3_^−^, mg/kg	NH_4_^+^, mg/kg	Cl^−^, mg/kg	CO_3_^2−^, mg/kg	Na^+^, mg/kg	Mn^2+^, mg/kg	Mg^2+^, mg/kg	K^+^, mg/kg	Fe^2+^ + Fe^3+^, mg/kg	Total organic carbon, %
S1	8.61	traces	1.06	4.5	51.92	129.29	297.8	161.1	720.8	376.7	1.41	0.04
A-6/99-6	8.21	traces	0.78	2.56	62.3	172.39	915.2	331.4	10.47	107.0	34.22	0.01

Xerosol sample S1 was sampled in mountain desert at the foot of Atlas mountain system, Morocco. At this region, the Ibn Battuta Centre (ESA) and NASA conduct tests of landing modules and scientific equipment for Martian missions with astrobiological tasks. The area is considered as a terrestrial natural analog of Mars [38],[39]. The main rock type is andesite. The territory is formed by the volcanic rise of the lower layers of the crust with the emergence of ancient deposits on the surface, dated with Precambrian—the Upper Paleozoic periods. The sample was taken from the A horizon at 5–10 cm depth. In the aqueous extract, the cations Mg^2+^ and K^+^ and the Cl^−^ and CO_3_^2−^ anions dominated (Table 1). Prior to experiment, the sample was stored sealed in an air-dry state at +4 °C temperature.

### 2.2. Samples preparation and irradiation

Prior irradiation, the S1 soil sample with weight ∼20 g was moistened with 5 mL sterile distilled water and incubated in a thermostat at a temperature of +28 °C without adding any reagents or substrates in a tightly closed sterile polypropylene container for 10 days in order to revive the microbial community, then dried again to an air-dry state for a day at the same temperature in the same container without cap but tightly covered with sterile chemically resistant non-woven wipes (Kimberly-Clark, USA). The same way but without adding water, activation of the microbial community in the permafrost sample with weight ∼20 g was provided. The moistening of that sample was occurred due to the ice melting. Preliminary tests shown that drying for a day is enough to reach air-dry moisture for these samples. The samples moisture was measured using MF-50 moisture analyzer (AnD, Japan). The moisture of the air-dried S1 and A-6/99-6 samples was 1.45% and 1.02%, respectively. For the irradiation, ∼5 g aliquots of the each sample were placed in the previously described climatic chamber [40], which allows maintain 1 Torr pressure (with the air composition in the chamber equivalent to the atmospheric air composition) and temperature −50 °C during the entire irradiation time. The climatic chamber is a forevacuum chamber with a zeolite cryogenic pump inside for effective capturing of the water vapour and other gases. The forevacuum chamber is surrounded by a jacket filled with liquid nitrogen. The chamber contains a cylinder about 1 cm in diameter and about 12 cm long divided on two similar parts, in which a both samples were placed simultaneously. The irradiation was carried out on K-120000 gamma facility with ^60^Co sources at 1 kGy/h radiation intensity for 40 hours. Radiation intensity was measured using Fricke dosimeters. Activated unirradiated samples were used as controls. Immediately after irradiation, the samples were placed at −18 °C and stored until analysis.

### 2.3. Culturing

Determination of culturable heterotrophic aerobic bacteria number was carried out by plating on solid nutrient glucose-peptone-yeast (GPY) media: (peptone: 2 g/l, glucose: 1 g/l, yeast extract: 1 g/l, casein hydrolyzate: 1 g/l, CaCO_3_: 1 g/l, agar-agar: 20 g/l) and ½ R2A (R2A agar (“Difco”, USA): 9.1 g/l, agar-agar: 15 g/l). Prior to inoculation, the soil suspension (1:100) with sterile distilled water was vortexed using Heidolph Multi Reax vortex for 30 minutes at 2000 rpm for microorganisms' desorption from the surface of mineral particles. Suspensions of samples in different dilutions were plated in triplicate. Simultaneously contamination controls were performed, in particular, plating of the water which was used for the dilutions preparation, exposure of the open Petri dishes to the air for air microorganism presence control, and incubation of dishes with the nutrient media to control medium sterility. The culturing was carried out at +28 °C temperature. The contamination controls were incubated along with the plated dishes.

The use of temperature +28 °C for culturing of microorganisms from Antarctic samples was justified by known data on prevalence of psychrotrophic and mesophilic heterotrophic bacteria among isolates from this region, and almost lack of psychrophilic strains [11],[41],[42]. In addition, preliminary tests revealed that the highest yield of bacteria and the greatest diversity of colonies' morphotypes in these permafrost samples manifested in the mesophilic temperature range.

It should be noted, that we used different method of microbial cells' desorption at culturing in comparison with epifluorescent microscopy (EFM), fluorescense *in situ* hybridization (FISH), and multisubstrate testing (MST) methods (see 2.4–2.6 sections). The vortexing at culturing was used because it should decrease the contamination risks compared to ultrasonic desorption. At desorption cells with ultrasound it is necessary to immerse the tip of the ultrasonic homogenizer into the soil suspension. During this stage, contamination from the homogenizer and from the air is possible. This stage is absent during vortexing. The EFM, FISH, and MST methods have lower detection limits, and due to this they are not so sensitive to contamination, therefore we did not modified samples pretreatment for these analysis. Preliminary study on comparison of ultrasound desorption and desorption with vortexing was performed. It was found, that number of cultured bacteria varied within the error of the method, and the similar diversity of the colonies morphotypes was observed.

### 2.4. Epifluorescence microscopy (EFM)

The total number of prokaryotes in the samples was determined with epifluorescence microscopy (EFM) with acridine orange dye. Cell desorption was carried out using an ultrasonic disintegrator at 22 kHz, 0.4 A, 2 minutes. The preparations were prepared in a six replicates, fixed by heating, then stained with water solution of acridine orange (1:10000) for 3 minutes, washed in standing water for 20 minutes, dried at the room temperature, and viewed under the Biomed-6 PR LUM (Russia) microscope with ×700 magnification for 20 fields of vision for each replication. Cells with a green fluorescence were counted. The water used for the dilution preparation was simultaneously examined as control. The prokaryotic cells number was calculated by the equation N = (S_1_ × a × n)/(V × S_2_ × c), where N—the number of cells per gram of soil; S_1_—the area of the preparation (µm^2^); a—number of cells in the field of view; n—the dilution index; V—the volume of the soil suspension drop placed to the glass (mL); S_2_—the field of view of the microscope (µm^2^); c—the sample weight (g).

### 2.5. Fluorescence in situ hybridization (FISH)

The number of *Bacteria* and *Archaea* in the samples was estimated using fluorescence *in situ* hybridization (FISH) method with rRNA-specific fluorescently labeled oligonucleotide probes. In the present work, the ARCH915 probe and a mixture of probes EUB338 + EUB338I (“Synthol”, Russia), specific for the representatives of the *Archaea* and *Bacteria* domains, respectively [43] were used. The assay was performed according to the protocol described earlier [43]. Preparations prepared in duplicate were examined on a ZEISS Mikroskop Axioskop 2 plus (Germany) fluorescent microscope with Filter set15 filters for 32 fields of view for each replication. The water used for the dilution preparation was simultaneously examined as control. Cells with red fluorescence were taken into account. The number of cells was calculated according to the equation N = (S_1_ × a × n)/(V × S_2_ × c), where N—the number of cells per gram of soil; S_1_—the area of the preparation (µm^2^); a—number of cells in the field of view; n—the dilution index; V—the volume of the cells' suspension drop placed on the glass (mL); S_2_—the field of view of the microscope (µm^2^); c—the sample weight (g).

It should be noted, that FISH method allows detection only metabolically active prokaryotic cells, while dormant cells are generally not detected by this method without the use of special techniques [44].

### 2.6. Multisubstrate testing (MST)

The potential metabolic activity of microbial communities in the samples was estimated on the basis of the consumption spectra of organic substrates (Table 2) by multisubstrate testing method. The method is an ecological modification of the well-known BIOLOG method used to investigate the functional activity of bacterial cultures for the purpose of express determination of their taxonomic affiliation [45]–[47].

**Table 2.** Sole carbon sources for the Multisubstrate Testing method.

Nominal group of substrates	Substrates
Pentoses	Arabinose, ribose, xylose
Hexoses	Glucose, fructose, rhamnose
Oligoses	Cellobiose, lactose, maltose, sucrose
Salts of carboxylic acids	Acetate, aspartate, citrate, succinate, maleinate, propionate, octanoate, lactate
Amino acids	Glycine, proline, leucine, norleucine, histidine, norvaline, treonine, alanine, asparagine, valine, serine, phenylalanine, glutamine, arginine, lysine, cisteine
Alcohols	Dulcitol, glycerol, inositol, sorbitol, mannitol
Polymers	Starch, Dextran 500, Tween 80, peptone, pullulan
Amides, Amines, Nucleosides	Creatinine, thymidine, carbamide

Soil aliquotes were placed in polypropylene tubes, filled with distilled water (1:100), and the cells were desorbed from mineral particles using ultrasound (22 kHz, 0.4 A, 2 minutes). Then the mineral particles were precipitated by centrifugation (2000 rpm, 2 minutes). The substrate consumption indicator (triphenyltetrazolium bromide) was added to the supernatant, and after mixing 200 µL aliquote was added to each well of a 96-well plate for immunological tests containing a set of 47 test substrates in duplicate (Table 2). The plates were incubated in a thermostat at +28 °C for 72 hours. After the incubation, the optical density of the solutions was measured photometrically at 510 nm wavelength, and the array of functional biodiversity indexes of the microbial community studied was calculated using the Eco-Log software [48],[49]. The water used for the dilution preparation was simultaneously examined as control.

### 2.7. Gas chromatography-mass spectrometry (GC-MS) of lipid markers

The taxonomic structure of microbial communities was determined by gas chromatography-mass spectrometry (GC-MS) of lipids. The analysis was carried out on the GC-MS system HP-5973 Agilent Technologies (USA) using the technique of multi-ion mass fragmentography of specific ions of mass spectra of genera- and species-specific higher fatty acids (FA) and cell wall aldehydes [50],[51].

The samples were dried in a thermostat at +40 °C. Aliquotes of dry sample was subjected to acidic methanolysis for an hour at +80 °C. As a result of this treatment, fatty acids were obtained in the form of methyl esters and fatty aldehydes in the form of dimethyl acetals (DMA), which were extracted with hexane. The hexane extract was dried and treated with a silylating agent. The obtained mixture of esters and DMA (2 µL) was introduced into a gas chromatography-mass spectrometry system injector. Chromatography was carried out in the temperature programming mode from +140 °C to +320 °C at a rate of 7 °C per minute.

To search for minor biogenic chemical components, mass-fragmentography method was used [50]. The program of multi-ion detection with accumulation of signals of 85 specific ions of fatty acids, aldehydes and sterols—marker substances of microorganisms was used. The mass spectrometer operated in a mode of periodic scanning from 15 to 20 ions in five time intervals. The intervals and ions were selected in way to selectively detect markers of identifiable species. The algorithm used to detect the mass spectral parameters of a biological sample allows to determine more than two hundred known FA, aldehydes and sterols of microorganisms, which is sufficient to identify and quantify more than 170 taxa of microorganisms at the level of the genus or species.

The areas of the markers' peaks on the mass-fragmentograms were integrated automatically and controlled manually by the standard programs of the gas chromatography-mass spectrometry system. The composition of microbial communities was calculated using Microsoft Office Excel electronic tables with the earlier developed calculation algorithm [52]. The substances in the chromatographic peaks were identified using library programs with NIST mass spectrometry databases.

### 2.8. Statistical analysis

Statistical processing of data was carried out using software packages STATISTICA 8.0, Microsoft Office Excel 2007, and Eco-Log software [48]. The results are presented with calculations of mean values and standard error of the mean (M ± m). The error is expressed as confidence intervals at p < 0.05. Cluster analysis of substrate consumption spectra by microbial communities was performed by Ward's method for 4 cases and 47 variables with 1-Pearson r linkage distance.

## 3. Results and discussion

### 3.1. The number and physiological state of Bacteria and Archaea

**Figure 1.**
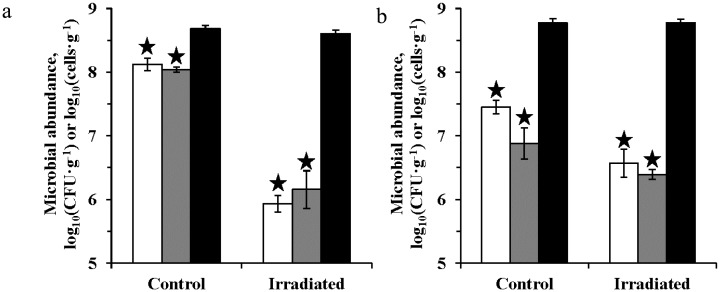
Effect of gamma radiation (40 kGy), low temperature (−50 °C), and low pressure (1 Torr) on the bacterial CFU count: (a) S1 sample; (b) A-6/99-6 sample. The number of CFU/g on PYG medium—white columns; the number of CFU/g on ½R2A medium—gray columns; the total number of prokaryotes (cells/g)—black columns. The error bars is within the confidence intervals at p < 0.05. Statistically different pairs of values are marked with asterisks.

After gamma irradiation with 40 kGy under low pressure (1 Torr) and low temperature (−50 °C), the number of proliferating bacterial cells in Antarctic permafrost sample (A-6/99-6) decreased by less than one order of magnitude, and in the sample of Xerosol soil from the Moroccan mountain desert (S1)—by 2 orders (Figure 1). Nevertheless, colony forming unit (CFU) counts in both samples remained at a sufficiently high level—3 × 10^6^ cells/g and 1 × 10^6^ cells/g, respectively. The contamination controls showed the absence of culturable contaminants in the nutrient medium, water used for dilutions, and in the laboratory air, which indicates that all cultured cells originated from the samples investigated. Contamination was not also detected at EFM, FISH, and MST analysis. The total numbers of prokaryotes *in situ* determined using epifluorescence microscopy (EFM) technique remained at the control level in both samples (Figure 1). We suppose that despite the reduction in CFUs numbers, microorganisms have transited into viable but nonculturable (VBNC) state under stress conditions [35].

FISH analysis revealed metabolically active *Archaea* and *Bacteria* in the samples. In Xerosol soil (S1), the number of active bacterial cells decreased 3.5 times after gamma irradiation, and the number of metabolically active *Achaea* increased almost 2-fold. Taking into account the EFM analysis data, which showed the preservation of the total abundance of prokaryotes at the control level, we should believe that the reason of bacterial cells' decreasing according to FISH is reducing of RNA synthesis due to transition of some bacterial populations to a resting state. The increased counts of *Archaea* after irradiation do not contradict this assumption. Irradiated samples were stored at −18 °C (about a month) before analysis. Doubling the number of *Archaea* during the storage is unlikely [3],[53]–[56]. However, temperature of −18 °C does not exclude metabolic processes. They could be increased in some archaeal populations that had not previously been detected using FISH method. At the same time, it can be hypothesized that samples' heterogeneity could be the reason for cells' numbers variations.

**Table 3.** Effect of gamma radiation (40 kGy), low temperature (−50 °C) and low pressure (1 Torr) on the ratio of metabolically active *Archea* and *Bacteria* and the relative content of metabolically active cells in prokaryotic communities *in situ*.

Sample	Archaea, %	Bacteria, %	Metabolically active cells fraction, %*
A-6/99-6—Control	22 ± 6	78 ± 4	58 ± 16
A-6/99-6—Irradiated	50 ± 9	50 ± 7	81 ± 10
S1—Control	17 ± 14	83 ± 6	99 ± 18
S1—Irradiated	57 ± 10	43 ± 8	63 ± 14

* is calculated as the ratio of the cumulative count of *Bacteria* and *Archaea* detected using FISH to the total number of prokaryotes detected using EFM.

**Figure 2.**
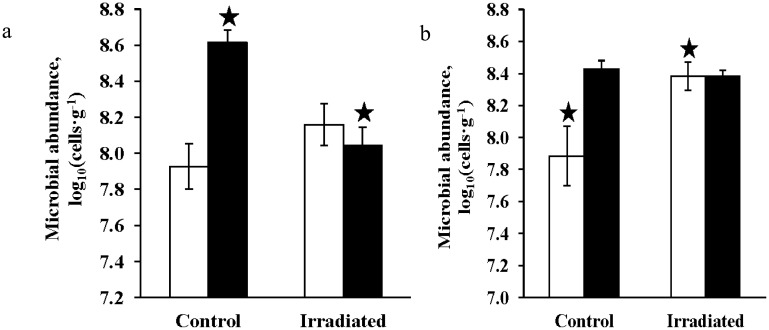
Effect of gamma radiation (40 kGy), low temperature (−50 °C), and low pressure (1 Torr) on the number of metabolically active cells of *Bacteria* and *Archaea*: (a) sample S1, (b) sample A-6/99-6; number of cells/g: archaea—white columns, bacteria—black columns. The error bars is within the confidence intervals at p < 0.05. Statistically different pairs of values are marked with asterisks.

In Antarctic permafrost (A-6/99-6) the number of metabolically active *Bacteria* detected using FISH method remained at the control level after irradiation at low pressure and low temperature. However, the number of metabolically active archaeal cells increased 3-fold (Figure 2). Total abundance of prokaryotic cells according to the EFM analysis did not change. Consequently, as in the variant with S1, the increasing number of *Archaea* can be explained by the change in metabolic activity of *Archaea* during storage or by the samples heterogeneity.

In accordance with the results obtained, it can be concluded that exposure to a high dose of gamma radiation in combination with low temperature and low pressure does not lead to the death of prokaryotic communities in soil or permafrost but can change their reproductive and probably metabolic activity. We suggest that some prokaryotes passed into VBNC or resting state, but confirmation of this requires a separate additional study. Metabolic activity of some populations probably can increase after irradiation at low temperature and low pressure. Thus, two types of reaction to stress are hypothesized in microbial communities *in situ*.

### 3.2. Functional state of microbial communities

**Figure 3.**
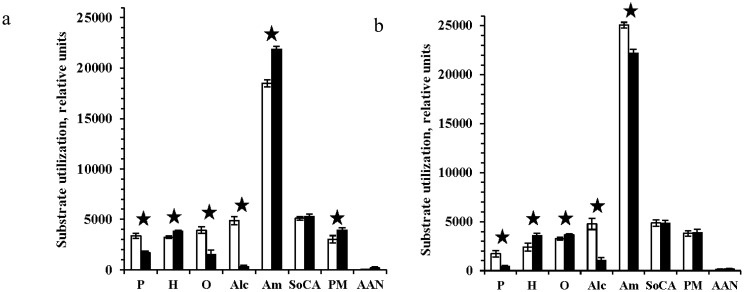
Substrates consumption by microbial communities: (a) sample S1, (b) sample A-6/99-6, before and after exposure to gamma radiation (40 kGy), low pressure (1 Torr) and low temperature (−50 °C). Control samples—white columns; irradiated samples—black columns. P: pentoses; H: hexoses; O: oligoses; Alc: alcohols; Am: amino acids; SoCA: salts of carboxylic acids; PM: polymers; AAN: amides, amines, nucleosides. Statistically different pairs of values are marked with asterisks.

Some integral parameters can give information concerning biodiversity and homeostasis of microbial ecosystem. These parameters changed insignificantly after irradiation and were close to the corresponding values of the control samples (Table 4). Metabolic activity varied identically in both irradiated samples: signs of rather rapid declining in the consumption of pentoses and alcohols occurred while marks of maintaining a high level of utilization of hexoses, amino acids, organic salts, and some polymers were observed (Figure 3).

**Figure 4.**
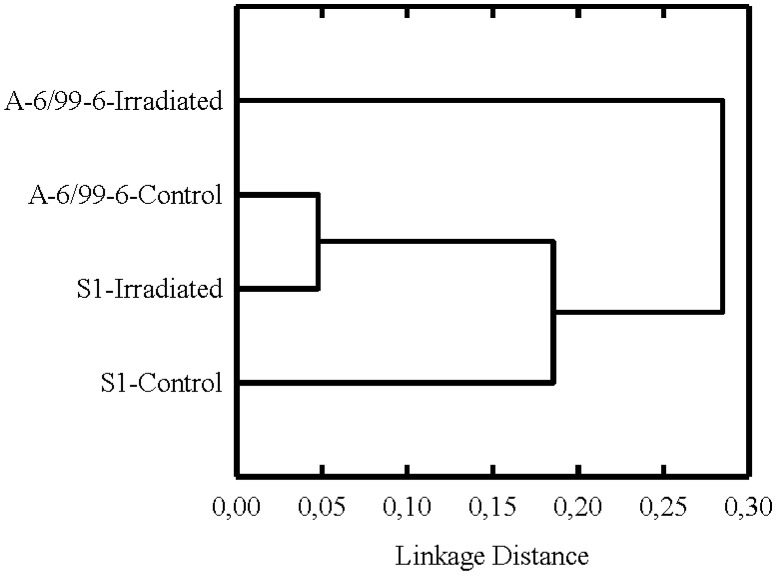
Cluster analysis (by Ward's method) of substrate consumption spectra by microbial communities of control and irradiated samples. Linkage distance: 1-Pearson r, n = 47.

Cluster analysis of data obtained demonstrates changes in metabolic fingerprint of microbial communities after irradiation (Figure 4). Both bacterial communities had identical changes in metabolic activity, and, subsequently, their spectra of substrates consumption were combined into cluster at the smallest distance. The changes were small, since the spectra of substrate consumption of all four samples have combined into a cluster at a short distance.

**Table 4.** Parameters of the functional diversity of bacterial communities before and after exposure to simulated conditions.

Sample	The coefficient of rank distribution of the spectra of substrates consumption, d	Number of substrates consumed, N	Specific metabolic work, W	Evenness, E	Shannon index, H	Integral indicator of the overall well-being of the system, G
A-6/99-6—Control	0.790 ± 0.125	25 ± 0	1992 ± 185	0.96 ± 0.01	4.47 ± 0.04	67.3 ± 10.3
A-6/99-6—Irradiated	1.139 ± 0.164	22 ± 0	1960 ± 224	0.96 ± 0.01	4.29 ± 0.05	41.1 ± 11.2
S1—Control	1.108 ± 0.138	27 ± 0	1897 ± 171	0.96 ± 0.02	4.57 ± 0.07	51.8 ± 7.1
S1—Irradiated	0.883 ± 0.186	25 ± 0	2047 ± 198	0.95 ± 0.01	4.30 ± 0.06	55.4 ± 8.8

In general, microbial communities of two ecotopes considered showed high resistance to radiation in model conditions, while retaining the initial cells' abundance, metabolic activity and functional diversity.

### 3.3. Microbial communities structure in situ investigation using GC-MC of lipid markers

Biodiversity of the microbial communities of Xerosol soil and permafrost has preserved at the control level after irradiation (Tables 5 and 6). The taxonomic composition of the microbial complexes remained practically unchanged, but quantitative changes of the lipid markers content of in the samples were observed.

**Table 5.** Number of cells (cells/g × 10^5^), characteristic for different bacterial groups, in samples of Xerosol soil according to GC-MS of lipids. Values in bold show out the most substantive changes after irradiation.

Group of bacteria	Sample	
	S1-Control	S1-Irradiated
*Actinomyces* sp.	50.13	42.65
*Mycobacterium* sp. I	13.20	17.21
*Nitrobacter* sp., *Hyphomicrobium* sp., *Xanthobacter* sp.	12.34	14.68
*Desulfovibrio* sp.	9.68	11.36
*Nocardia* sp., *Acetobacterium* sp.	9.58	10.06
*Deinococcus radiophilus*, *Methylococcus* sp.	**9.24**	**90.44**
*Streptomyces* sp., *Nocardiopsis* sp.	8.68	9.63
*Bacillus* sp.	7.24	6.03
*Mycobacterium* sp. II	**6.97**	**0.00**
*Hymenobacter* sp., *Ferric-reducing bacteria*	6.01	7.70
*Rhodococcus equi*	4.72	9.75
*Deinococcus maricopensis*, *Pseudonocardia* sp.	**4.04**	**18.94**
*Rhodococcus terrae*	3.03	7.56
*Clostridium omelianskii*, *С. pasterianum*, *C. acetobutyricum*	**1.83**	**24.09**
*Enterobacteriaceae*	3.39	3.94
*Caulobacter* sp.	3.07	3.70
*Micrococcus* sp., *Arthrobacter* sp.	2.96	3.79
*Corynebacterium* sp.	2.38	2.21
*Ruminococcus* sp., *Protozoa*	2.34	1.98
*Nocardiopsis* sp.	2.19	2.20
*Rhodobacter* sp.	**1.96**	**5.66**
*Acetobacter* sp., *Rhodobacter* sp.	1.86	2.27
*Bacillus subtilis*	1.63	4.71
*Actinomadura roseola*	1.59	1.69
*Pseudomonas freudenreichii*	1.47	3.97
*Nocardia carnea*	1.31	1.76
*Carboxydotermus* sp.	**1.29**	**0.00**
*Butyrivibrio* sp. 7S-14-3	1.20	1.33
*Pseudomonas putida*	0.74	1.10
*Sphingomonas capsulata*	0.59	1.02
*Sphingobacterium spiritovorum*	0.58	0.79
*Sphingomonas adgesiva*	**0.56**	**1.83**
*Streptococcus mutans*	0.47	0.56
*Eubacterium lentum*	0.29	1.09
*Wolinella* sp., *Acholeplasma* sp., *Roseomonas* sp., *Burkholderia* sp.	**0.29**	**1.38**
*Pseudomonas fluorescens*	0.25	0.44
*Butyrivibrio* sp. 1-2-13	**0.19**	**0.00**
*Pseudomonas vesicularis*	0.11	0.11
*Bacteroides ruminicola*	**0.10**	**0.00**
*Clostridium perfringens*	**0.08**	**0.50**
*Bifidobacterium* sp.	0.06	0.07
*Butyrivibrio* sp. 1-4-11	**0.04**	**0.16**
*Hymenobacter* sp., *Pontibacter* sp.	**0.03**	**0.63**
*Eubacterium* sp.	0.02	0.03

Minor changes in the lipid markers concentrations of most bacterial groups can be explained by the heterogeneity of the samples. However, after irradiation the content of lipid markers of *Deinococcus* spp., *Methylococcus* sp., *Pseudonocardia* sp., *Clostridium* spp., and *Rhodococcus* spp. has much increased in the S1 soil sample (Table 5), and concentrations of *Methylococcus* sp., *Clostridium* sp., *Sphaerotilus* sp., and *Rhodococcus* spp. lipid markers has distinctly rose in the A-6/99-6 permafrost sample (Table 6). It is known that representatives of these bacterial genera have high radioresistance [57]–[60]. As mentioned above, the samples were stored at a low temperature after irradiation, and a significant multiplication of bacteria during storage is unlikely. The most likely explanation for the increase in the concentration of a number of the some lipid markers is the change in the lipid composition of the bacterial cell walls as response to storage at low temperatures [61],[62]. This explanation seems plausible, given that the bacteria retained metabolic activity after irradiation (see Section 3.1).

The study of the microbial communities structure *in situ* using GC-MC analysis of lipid markers revealed that the combined effect of gamma irradiation with 40 kGy dose, low pressure (1 Torr), and low temperature (−50 °C) did not reduce the biodiversity of microbial communities inhabiting extreme ecotopes of the Earth.

### 3.4. Implications for the preservation of subsurface life on Mars

Microbial communities of samples from extreme habitats of different genesis and located in different climatic zones of the Earth showed high resistance to ionizing radiation with 40 kGy under conditions of low temperature and low pressure, retaining high number of viable cells, metabolic activity, functional integrity, and biodiversity.

The radiation resistance limit of microorganisms is considered about 20–25 kGy of gamma radiation, and the list of species withstanding such high radiation doses is short [57],[58],[63],[64]. In our study, high biodiversity of prokaryotes survived irradiation *in situ* in soil and permafrost samples. The data obtained can be explained by the decrease in radiation damage as the temperature during irradiation is lowered [21], or by decreasing water content (one of the main sources of free radicals) in samples, and possibly in cells, due to low pressure [16]. An additional contribution to the increase in radio-resistance of microorganisms *in situ* can also be made by other factors considered in the introduction to this article.

**Table 6.** Number of cells (cells/g × 10^5^), characteristic for different bacterial groups, in samples of Antarctic permafrost according to GC-MS of lipids. Values in bold show out the most substantive changes after irradiation.

Group of bacteria	Sample	
	A-6/99-6-Control	A-6/99-6-Irradiated
*Acetobacterium* sp. II	30.00	19.12
*Nitrobacter* sp.	28.56	19.75
*Mycobacterium* sp.	21.73	14.01
*Methylococcus* sp., *Clostridium* sp	**14.86**	**47.28**
*Arthrobacter globiformis*	6.23	3.73
*Corynebacterium* sp.	4.34	3.75
*Nocardia carnea*	3.54	2.70
*Ruminococcus* sp.	3.40	3.41
*Clostridium difficile*	3.26	1.84
*Sphaerotilus* sp.	**2.19**	**9.90**
*Staphylococcus* sp.	2.01	1.67
*Sphingomonas capsulata*	2.01	1.97
*Streptomyces* sp.	1.93	0.80
*Acetobacter* sp., *Cyanobacteria*	1.83	2.74
*Rhodococcus terrae*	**1.70**	**6.89**
*Caulobacter* sp., *Enterobacteriaceae*	**1.56**	**0.00**
*Bacillus* sp., *Cellulomonas* sp.	1.51	0.61
*Rhodococcus equi*	1.50	2.38
*Pseudonocardia* sp.	1.14	1.87
*Micrococcus* sp.	1.07	1.59
*Actinomadura roseola*	0.92	0.45
*Peptostreptococcus* sp.	**0.88**	**3.34**
*Cyanobacteria*	0.75	0.84
*Aeromonas hydrophila*	0.62	0.80
*Acetobacterium* sp. I	0.48	0.24
*Eubacterium lentum*	**0.39**	**0.00**
*Pseudomonas putida*	**0.29**	**1.00**
*Pseudomonas fluorescens*	0.18	0.31
*Pseudomonas vesicularis*	0.10	0.16
*Clostridium perfringens*	**0.07**	**0.26**
*Bacillus subtilis*	**0.00**	**0.85**
*Desulfovibrio* sp.	**0.00**	**0.60**
*Bacteroides ruminicola*	**0.00**	**0.35**
*Agrobacterium radiobacter*	**0.00**	**0.10**

Based on data on the radiation intensity at the Martian surface of 0.076 Gy/year [65], our results allow approximately to calculate the longevity of possible cryoconservation of putative microbial communities in the shallow layer of Mars' regolith (protected from UV radiation) for at least 500 thousand years after Mars lost a significant part of the atmosphere. With increasing depth, this time should increase. According to the calculations, at a depth of 20 cm, the radiation intensity is 45–65 mGy/year [66],[67]. In this case, the radiation dose of 40 kGy is accumulated by regolith within 600–900 thousand years. At a depth of 5 meters, it is possible to assume the conservation of a potential ancient biosphere for at least 8 million years with an irradiation intensity of 5 mGy/year [67].

The age of Martian permafrost is estimated at approximately more than 3 Ga [31]. It indicates, that survivability after irradiation with doses which accumulates during several millions years is not enough for viable cryopreservation of the putative ancient biosphere till present. At the current level of ionizing radiation intensity, the cryopreserved cells should accumulate ∼230 MGy and ∼15 MGy doses in the shallow layer of the regolith and at 5 meters depth, respectively. But early Mars probably had dense atmosphere, which could effectively shield cells against cosmic radiation [66]. Though the atmospheric pressure on the early Mars and the time of its decline are difficult to estimate, Martian atmosphere could protect putative biosphere during prolonged time. In addition, it should be noted, that 40 kGy dose was not sterilizing in our study, while sterilizing dose may be significantly higher. Definition of the limit for the microbial radioresistance in different extraterrestrial conditions is in the scope of the further research. Nevertheless, viable cryopreservation of putative ancient biosphere over 3 Ga without cells replication or metabolism (i.e. without radiation damages repairing) seems unlikely. However, there are evidences of mild climate and the presence of liquid water in last hundreds of thousands and few millions years on the Mars [67]–[70]. Microorganisms should survive in the permafrost sediments of such ages even at shallow depths.

The calculations presented above do not take into account the possibility of repairing damages by cells at low temperatures. However, it is known that microorganisms are able to multiply at temperatures down to −18 °C and maintain metabolic activity at −33 °C [3],[71]. Taking into account these data, we can assume an even longer preservation of the potential biosphere of Mars. In some areas on Mars, temperatures above 0 °C and formation of liquid water are possible [3],[40],[72]–[74]. In such a case, quick repairing and reproduction of cells could be assumed, with subsequent preservation in the dormant state until the next period of favorable environmental conditions [4]. To date, it is shown that microorganisms are able to grow under combined low pressure, temperature, and anoxic atmosphere conditions [75], well withstand impact of Martian salts and strong oxidizers [76]–[79], can survive in subzero brines [80],[81]. If favorable conditions for microorganisms' growth and metabolism are created more often than every 500 thousands to 8 million years, the duration of viable cryoconservation of the putative biosphere of Mars can be practically unlimited.

Presented findings also contribute to the assessment of the risk of Mars or the Earth contamination with the past and future missions. Results of the study indicates that putative contaminants are able to survive in a shallow regolith layer (protected from ultraviolet) for thousands years. It is important, that high resistance *in situ* is characteristic for bacterial communities with wide bacterial diversity, and not only for the particular extremophilic species. It is especially significant in the light of the detection of diverse microbial communities in cleanrooms in which ExoMars 2016 mission was assembled [82]. Furthermore, data discussed evidence possibility of microorganisms' growth and replication under present Martian conditions. Contaminants from shallow regolith might be widely redistributed by the dust storms [21], and it could significantly complicate further search of the native life on the planet. From the microbiological point of view, it is impossible to ensure full sterilization of landers, but it is necessary to improve methods for reducing microbial load on space missions. In particular, the protocols can include new sterilization techniques [82],[83] and antibacterial surfaces [84]. Since the study presented testifies in favor of life existence possibility on the Mars, it should be taken into account at sample return missions planning meaning appropriate protocols development and implementation to prevent Earth contamination.

### 3.5. Uncertainties and limitations

We were not able to reproduce the gas composition of the Martian atmosphere. The pressure and composition of the atmosphere can affect the radiation damage in two ways: (a) through the desiccation of the sample and cells with a decrease in pressure, and, consequently, a decrease in the concentration of water, which is one of the main sources of free radicals [15],[16], (b) over the concentration of oxygen in the atmosphere, which also is one of the main sources of free radicals [16]. The pressure created was close to the atmospheric pressure on the surface of Mars [1],[3]. Thus, we probably reproduced the desiccation processes quite accurately. However, the climatic chamber used to model the surface conditions of Mars did not allow us to reproduce the gas composition of the Martian atmosphere. Thus, in our experiment there was a somewhat greater concentration of oxygen in the atmosphere than on Mars. However, when the pressure was reduced to 1 Torr we removed ∼99.9% oxygen, and the remaining ∼0.01% probably could not significantly affect the radiation effects.

The salts concentrations in our samples (Table 1) do not reproduce the ones found in Martian regolith [85]–[87]. We also believe that mineral composition was not reproduced too. Obviously, regolith's chemistry should vary in space as well as at microscales [87]–[89]. It should create a diversity of niches with different chemical characteristics. In concentrations found in Martian regolith salts in itself do not strictly limit bacteria survival [76]–[81], and can even promote the condensation of liquid water by cells [80]. At the same time, salts and minerals may be sources of free radicals at radiolysis [90]. While the water and oxygen are considered as the most important sources of free radicals [15]–[17], contribution of salts and mineral matrix is unclear. As far as we know there are no studies on salts presence effect on bacteria survival under ionizing irradiation. Recently effects of pretreatment and post treatment of irradiated bacteria with salts was studied, but there were no clear patterns found [77],[78]. As was mentioned above, there are some data on the influence of organo-mineral matrix composition on irradiation effects, but these data are incomplete and contradictory [24]–[26]. Issues discussed should be further investigated.

The ionizing radiation in the Martian regolith consists of protons, electrons, pions, muons, ions, etc. [66]. These components have different effects on biological objects, and their effects also differ from gamma-radiation impact [16]. However, the sources of ionizing particles are limited in the particle fluence as well as in the irradiated sample volume due to narrow beam sizes and small penetration depth [21]. Due to this the gamma and X-ray sources are commonly used in space radiobiology studies [18],[21],[63],[78]. These sources offer a high irradiation intensity, high penetration, and practicality of experimentation, even though it is taken into account that the ionization and, consequently, some biological effects it causes do not fully reproduce the effects of accelerated particles [21]. Moreover, with the regolith depth increasing the weakly ionizing secondaries of the soft core of the cosmic ray cascades, such as muons, electrons, and gamma rays, begin to dominate by 2 m depth. Therefore, the simulation of the cosmic rays by gamma irradiation becomes more accurate with depth increasing [21],[66].

The irradiation intensity used in the experiment is by the several orders of magnitude higher than on the present Mars [65],[66]. Obviously, it is impossible to irradiate samples for thousands and millions years, according to common practice in related studies [18],[21],[63],[78]. The irradiation intensity used in present study is compatible with the regimes of other similar studies. It is known, that in general the effectiveness of a sparsely ionizing radiation (including gamma-radiation) dose is reduced with irradiation intensity decreasing [16]. Usually it is explained by recovery phenomena. In the earlier study on bacteria survival under gamma-irradiation at −79 °C several radiation rates were used, but there was not clear differences observed [21]. It can be explained by low rate or full stop of metabolism under low temperature conditions [3]. But considering Mars putative biosphere it should be taken into account the long time of irradiation. If even slow repairing of cells' damages occurs, it can be of great effect in the course of geological time. As was discussed above, there are hypothetic possibilities for such reparation and even periodically for growth and replication. Furthermore, progressive increasing of microorganisms' radioresistance can be hypothesized taking into account data on directed evolution of radioresistance in bacteria under impact of sublethal doses of ionizing radiation [91],[92]. Summarizing presented facts and assumptions regarding the irradiation intensity it can be concluded that in situation of Mars the prolongation of viable cells' *in situ* cryoconservation should occur in comparison with our model experiment evaluation.

In general, taking into account the uncertainties and limitations discussed, we believe that we have reproduced a set of the most important physical factors influencing radioresistance. At the same time in the scope of our further research is development some more accurate models, meaning salts and oxidizers addition, as well as study of different ionizing radiation type's effects. Furthermore, the definition of the sterilizing dose of radiation is one of the most important tasks.

## 4. Conclusions

Viability of microbial communities of ancient permafrost (Dry Valleys, Antarctica) and Xerosol soil (mountain desert in Morocco) was studied after gamma-irradiation with 40 kGy dose at low pressure (1 Torr) and low temperature (−50 °C) conditions. Microbial communities inhabiting these samples showed *in situ* high resistance to the applied effects, retained high number of viable cells, metabolic activity, and high biodiversity. Based on the results, it is assumed that the putative ancient Martian biosphere could be viable cryopreserved for at least 500 thousand years and 8 million years in the surface layer of Mars regolith and at 5 m depth, respectively, in the dormant state with the current level of ionizing radiation intensity.
